# Butterfly gliomas: a time for stratified management?

**DOI:** 10.1007/s10143-023-02126-w

**Published:** 2023-09-04

**Authors:** Siddharth Sinha, Adam Avnon, Andrea Perera, Jose Pedro Lavrador, Keyoumars Ashkan

**Affiliations:** 1 Department of Neurosurgery, King’s College Hospital NHS Foundation Trust, London, UK; 2 Maurice Wohl institute, Kings College London, London, UK; 3 Institute of Psychiatry, Psychology & Neuroscience, King’s College London, London, UK

**Keywords:** Butterfly glioblastoma, Corpus callosum, Transcallosal, Radiotherapy, Chemotherapy

## Abstract

Butterfly glioblastomas (bGBM) are a rare subset of WHO grade IV tumours that carry a poor prognosis with a median survival ranging between 3.3 to 6 months. Given their poor prognosis, there is debate over whether histological diagnosis with a biopsy or any surgical or oncological intervention alters disease progression. With this in mind, we reviewed our experience as a high-volume unit to evaluate management decisions and outcomes. A retrospective analysis was undertaken (January 2009 to June 2021) of the electronic patient records of a large neurosurgical centre. We assessed patient demographics, initial clinical presentation, tumour characteristics, clinical management and overall survival (Kaplan–Meier estimator, log-rank analysis and cox proportional hazard analysis). Eighty cases of bGBM were identified. These patients were managed with biopsy ± adjuvant therapy (36), with radiotherapy alone without biopsy (3), or through surgical resection (3). Thirty-eight cases of suspected bGBM were managed conservatively, receiving no oncological treatment or surgical resection/biopsy for histological diagnosis. Those managed conservatively and with radiotherapy without biopsy were diagnosed at neuro-oncology multidisciplinary meeting (MDT) based on clinical presentation and radiological imaging. No significant difference in survival was seen between conservative management compared with single adjuvant treatment (*p* = *0.69*). However, survival was significantly increased when patients received dual adjuvant chemoradiotherapy following biopsy or resection (*p* = *0.002*). A Cox Proportional Hazards model found that survival was significantly impacted by the oncology treatment (*p* < 0.001), but was not significantly related to potential confounding variables such as the patient’s age (*p* = 0.887) or KPS (*p* = 0.057). Butterfly glioblastoma have a poor prognosis. Our study would suggest that unless a patient is planned for adjuvant chemoradiotherapy following biopsy, they should be managed conservatively. This avoids unnecessary procedural interventions with the associated morbidities and costs.

## Introduction

Glioblastoma multiforme (GBM) is an aggressive, grade IV [1], central nervous system cancer that typically spreads along white matter tracts. A rare subset of these, butterfly glioblastoma (bGBM), arises when they extend along the corpus callosum and into the two hemispheres—the epithet ‘butterfly’ refers to the characteristic shape that such tumours produce on magnetic resonance imaging [2–4]. bGBM have a median survival ranging between 3.3 and 6 months [5].

bGBM represents a more aggressive entity than other radiologically focal GBM tumours, and this is represented by a reduction in expected survival. This is correlated by the molecular hallmarks of bGBM compared to other subsets of GBM. Boaro et al. reports IDH (isocitrate dehydrogenase) mutation, a favourable prognostic indicator to be lower (3.8%) in bGBM than GBM (5–12%), as well as bGBM having lower MGMT (O6-methylguanine-DNA-methyltransferase) methylation rates than GBM, although the latter was not significant [4, 6].

Due to the poor prognosis associated with bGBM and the invasive nature of the disease, along with the morbidities associated with prolonged surgery, surgical resection is controversial, with most patients only undergoing a biopsy [4, 5]. Chojak et al. conducted a meta-analysis of five studies with 194 patients with bGBM [5] (Table 1). This study assessed mortality at 6, 12, and 18 months of patients undergoing surgical resection versus biopsy only. At 6 months, mortality was decreased in those undergoing surgical resection compared to biopsy (Relative risk (RR) 0.63 [95% CI 0.44–0.91]). However, no significant difference was observed with overall survival at 12 months (RR 0.76 [95% CI 0.50–1.14]) and 18-months (RR 0.84 [95% CI 0.56–1.26]). Chojak et al. did find substantial heterogeneity between the 5 datasets within the meta-analysis at 12 and 18 months outcomes. Therefore, a sensitivity analysis was conducted in which each individual study was removed followed by a recalculation of RR [5]. The sensitivity analysis found that the exclusion of Chaichana et al. favoured resection in the 4 remaining studies (RR 0.68 [95% CI 0.48–0.97]) [5, 6].

**Table 1:** Studies assessing management of bGBM

Study	Country	Recruitment period	Total	Resection	Biopsy
			*N*	*N*	*N*
Franco et al. 2021	Germany	2005–2017	55	25	30
Chaichana et al. 2014	USA	2007–2012	48	29	19
Dayani et al. 2018	USA	2004–2014	39	14	25
Dziurzynski et al. 2012	USA	2000–2010	23	11	12
Opoku-Darko et al. 2018	Canada	2004–2016	29	9	20
Boaro et al. 2021	USA	2008–2018	62	26	36

In addition, Boaro et al. also found that surgical resection of bGBM had a greater median overall survival (OS) when compared to biopsy, 11.5 months (95%CI 7.7–18.8) versus 6.3 months (95%CI 5.1–8.9) respectively [4]. Both Chojak and Boaro et al. report the use of adjuvant therapy being associated with improved survival particularly in those patients who underwent surgical resection [4, 5].

Here, we aimed to assess the impact of the extent of resection and the adjuvant therapy regimen on the outcome of patients with bGBM. We further analysed the impact of a range of factors, not previously well studied in this cohort of patients, such as the size of the tumour, location along the carpus callosum and the molecular signatures (IDH and MGMT methylation status).

## Methods

### Patient data

We performed a retrospective analysis from January 2009 to June 2021 of our electronic patient records to identify all patients with a primary presentation of a grade IV bGBM through multidisciplinary team (MDT) records. We assessed patient demographics, initial clinical presentation, tumour characteristics (location, mutation, volumetric size), clinical management and overall survival (Kaplan–Meier estimator log-rank analysis and cox proportional hazard analysis).

Our inclusion criteria allowed for both adult and paediatric patients with a primary presentation of a grade IV glioblastoma which were centred on the corpus callosum, extending to both hemispheres. The exclusion criteria included tumour reoccurrence presentations, grade I–III gliomas and other intracranial lesions.

Volumetric assessment was conducted based on MRI head imaging prior to treatment using Stealth Station Surgical Navigation (Medtronic). Each patient had the preoperative tumour size and brain size measured, followed by calculation of brain/tumour ratio. The ‘seed’ tool was used to estimate tumour size utilising all planes.

### Statistical analysis

Patient data was analysed utilising Microsoft excel and survival curves were analysed utilising International Business Machines Corporation (IBM) Statistical Package for the Social Sciences software (SPSS). A value of *P* < 0.05 was considered to be statistically significant.

## Results

**Fig. 1:**
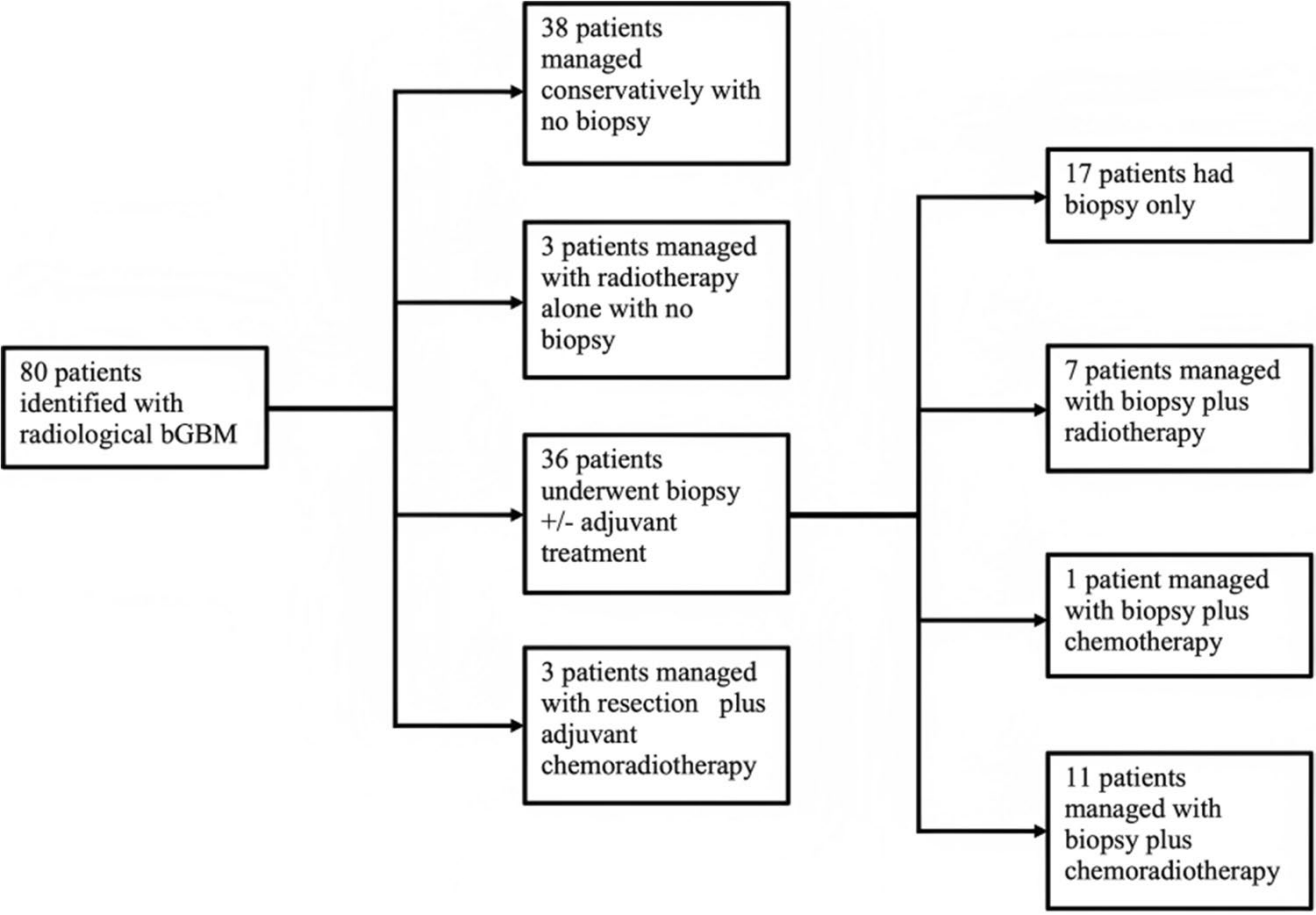
Patient management flowchart

Between January 2009 and June 2021, 80 cases of bGBM were identified. These patients were managed with biopsy ± adjuvant therapy (36), with radiotherapy alone without biopsy (3), or with surgical resection (3). Thirty-eight cases of suspected bGBM were managed conservatively: receiving no oncological treatment, biopsy or tumour resection (Fig. 1). Those managed conservatively and with radiotherapy without biopsy were diagnosed at MDT based on clinical presentation and radiological imaging.

### Patient demographics

The median age of all patients was 67 (ranging from 17 to 89), 46 male patients (57.5%) and 34 female patients (42.5%). The most common presenting complaints were headache and confusion (29 patients, 36.3% each), followed by motor deficit (22 patients, 27.5%) and gait disturbance (21 patients, 26.3%) (Table 2).

**Table 2:** Patient demographics

	All bGBM patients	Conservative	Radiotherapy alone	Biopsy	Resection	Univariable p value
Number	80	38	3	36	3	
Sex (%)						0.161^a^
Females	34 (42.5)	16 (42.1)	3 (100)	13 (36.1)	2 (27.3)	
Males	46 (57.5)	22 (57.9)	0 (0)	23 (63.9)	1 (48.3)	
Age at diagnosis						
Median (Range)	67 (17–89)	77.5 (49–89)	77 (57–85)	60 (31–71)	61 (17–67)	< 0.001* ^b^
Karnofsky Performance Status						
Median (Range)	80 (20–100)	70 (20–90)	70 (50–90)	90 (40–100)	NA	0.010* ^a^
Presenting symptom (%)						
Motor deficit	22 (27.5)	11 (28.9)	0 (0)	10 (27.8)	1 (33.3)	0.839 ^a^
Sensory deficit	5 (6.3)	2 (5.3)	0 (0)	3 (8.3)	0 (0)	0.790 ^a^
Gait instability	21 (26.3)	9 (23.7)	1 (33.3)	11 (30.6)	0 (0)	0.709 ^a^
Visual	6 (7.5)	1 (2.6)	0 (0)	4 (11.1)	1 (33.3)	0.238 ^a^
Headache	29 (36.3)	7 (18.4)	2 (66.7)	19 (52.8)	1 (33.3)	0.018* ^a^
Confusion	29 (36.3)	15 (39.5)	3 (100)	10 (27.8)	1 (33.3)	0.053 ^a^
Seizure	9 (11.3)	4 (10.5)	0 (0)	5 (13.9)	0 (0)	0.645 ^a^
Other	47 (58.8)	21 (55.3)	1 (33.3)	24 (63.9)	2 (66.7)	0.755^a^
Location (%)						0.737^a^
Genu/Rostrum	40 (50.0)	17 (44.7)	1 (33.3)	19 (52.8)	3 (100)	
Body	10 (12.5)	6 (15.8)	1 (33.3)	3 (8.3)	0 (0)	
Splenium	23 (28.8)	12 (31.6)	1 (33.3)	10 (27.8)	0 (0)	
Genu/body	2 (2.5)	1 (2.6)	0 (0)	1 (2.8)	0 (0)	
Splenium/body	5 (6.3)	2 (5.3)	0 (0)	3 (8.3)	0 (0)	
IDH status (%)						1.000^c^
Wildtype	33 (41.3)	NA	NA	30 (81.1)	3 (100)	
Mutant	1 (1.25)	NA	NA	1 (2.7)	0 (0)	
NA	46 (57.5)	NA	NA	5 (13.5)	0 (0)	
MGMT promoter status (%)						1.000^c^
Unmethylated	12 (15)	NA	NA	11 (29.7)	1 (33.3)	
Methylated	19 (23.8)	NA	NA	17 (45.9)	2 (66.7)	
NA	49 (61.25)	NA	NA	8 (21.6)	0 (0)	
Pretreatment tumour volume, cm^3^						0.256^b^
Median (Range)	53.50 (19.9–122.2)	46.20 (30.5–57.6)	37.65 (37.6–37.7)	57.60 (19.9–122.2)	98.00 (NA)	
Pre-treatment brain volume, cm^3^						0.220^b^
Median	1225.80 (847.5–1646.9)	1271.60 (987.2–1405.8)	937.15 (847.5–1026.8)	1225.80 (878.2–1646.9)	1223.50 (NA)	
Tumour/brain ratio						0.265^b^
Median	0.04 (0.01–0.11)	0.04 (0.02–0.05)	0.04 (0.04–0.04)	0.05 (0.01–0.11)	0.08 (NA)	
Adjuvant therapy (%)						
Radiotherapy	10 (12.5)	NA	NA	7 (19.4)	0 (0)	
Chemotherapy	1 (1.3)	NA	NA	1 (2.8)	0 (0)	
Chemoradiotherapy	14 (17.5)	NA	NA	11 (30.6)	3 (100)	
No adjuvant	55 (68.8)	NA	NA	17 (47.2)	0 (0)	
Survival outcomes (months)						
OS median (95%CI)	2.8 months (2.1–3.4)	2.2 months (1.6–2.8)	2.3 months (0.8–3.9)	3.6 months (1.7–5.6)	11.8 months (1–22.6)	0.042*^d^
Deaths (%)	73 (91.3)	36 (94.7)	3 (100)	31 (86.1)	3 (100)	

Chi-squared (Likelihood ratio)^a^, Kruskal–Wallis test^b^, Fisher-exact test^c^ and Kaplan-Meier^d^

There was no significant difference in gender between groups. There was a significant difference in age distribution (*p* < 0.001) between each group with those in the conservative and radiotherapy group having a median age of 77.5 and 77 respectively, while those in the biopsy and resection group having median ages of 60 and 61 respectively. When assessing symptoms, headache was the only symptom which was found to significantly vary between groups (*p* = *0.018*) occurring most often within the biopsy group (*n* = 19, 52.8%). No significant difference was detected in tumour or brain size pretreatment between groups (Table 2).

Karnofsky performance status (KPS) was found to be significantly different between groups (*p* = *0.010*). The median KPS was 70 in the conservative and radiotherapy group compared to 90 in the biopsy group. The preoperative KPS was only available for 1 patient in the resection group (90).

### Radiological appearance and histology

Tumour infiltration was seen across the corpus callosum with 50% in the Genu (40), 12.5% in the body (10), and 28.8% in the Splenium (23). Two patients had tumour infiltration between the Genu and body and 5 patients between the body and the splenium. The median pretreatment tumour volume was 53.50cm^3^, with the median brain volume 1225.8cm^3^. The median tumour to brain ratio was 0.04 in all patients (Table 2).

From all patients who underwent histological diagnosis, IDH1 mutation was found in 1 patient and methylated MGMT present in 19 patients.

### Surgery

Surgical resection was advised by the neuro-oncology multidisciplinary team in 3 patients (Table 3). The reasoning ranged from the premorbid status of the patients to risk of tumour causing acute hydrocephalus. To elaborate, one patient was offered surgery given the young age of 17 and excellent performance status, whilst the other 2 patients had impending hydrocephalus and were thus offered debulking surgery but with gross total resection achieved as per the operative notes. All these 3 patients had postoperative chemotherapy and radiotherapy, with no complications occurring post-operatively. The rationale for surgically managing these patients varied.

**Table 3:** Operative information for the patients who underwent surgical resection

Surgical patients	Age	Reason for operation	Operation title	Operation date	Post operative complications
1	17	Close proximity of lesion to the foramina of munro. Resection was decided to reduce risk of acute hydrocephalus	Right parasagittal craniotomy for tumour	17/10/2013	No complications reported postoperatively
2	61	Peripheral proximity of the lesion and good premorbid status	Stealth guided right frontal craniotomy for tumour	24/10/10	No complications reported postoperatively
3	67	Good premorbid status	Right frontal mini craniotomy/excision of tumour	27/10/11	Reduced attention reported postoperatively

### Complications

Of the 36 patients who underwent biopsy, postoperative complications occurred in 4 patients including 1 deep vein thrombosis, 1 chest infection and 1 drop in GCS resulting in a short admission to intensive care. The final complication related to a patient who continued to deteriorate post-operatively with worsening performance status, requiring palliative care input, who passed away while an inpatient.

### Survival analysis

**Fig. 2:**
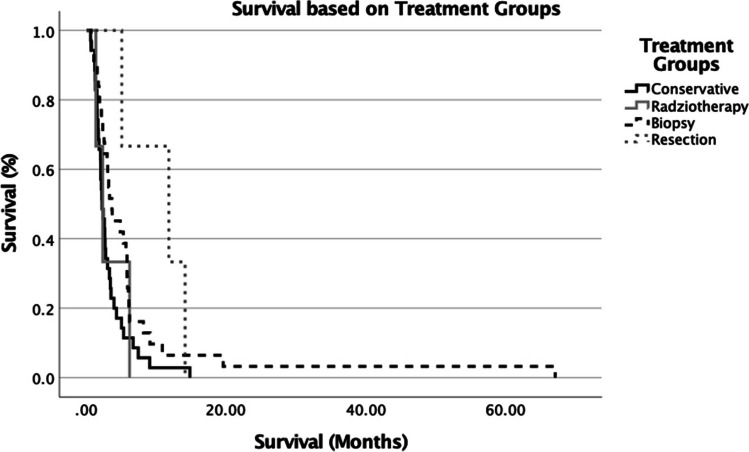
Survival across main treatment groups (Kaplan–Meier log-rank curve) (*p* = *0.042*)

**Fig. 3:**
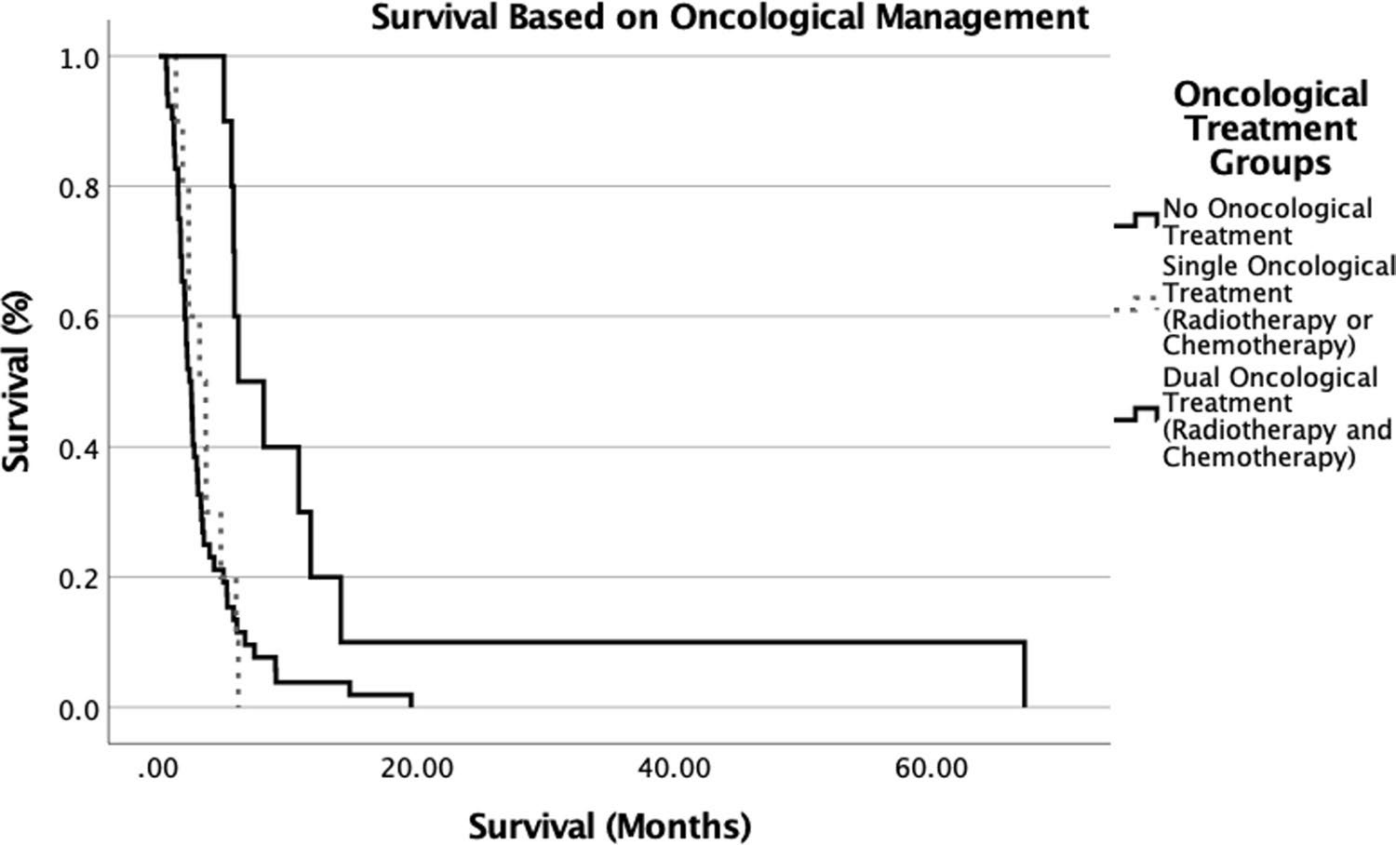
Survival across oncological treatment groups (Kaplan–Meier log-rank curve) (*p* = *0.002*)

At the time of data analysis, 91.3% of patients were deceased following diagnosis with a bGBM with median overall survival (OS) of 2.8 months (95% confidence interval [95%CI], 2.1–3.4 months). Patients who underwent a surgical resection had the longest median OS of 11.8 months (95%CI, 1–22.6 months) followed by 3.6 months (95% CI 1.7–5.6 months) for biopsy ± adjuvant treatment (Table 2). Conservative management and radiotherapy alone had a median OS of 2.2 months (95% CI 1.6–2.8 months) and 2.3 months (95% CI 0.8–3.9 months) respectfully (Table 2). When comparing these 4 group there was a significant difference in survival (*p* = *0.042*) (Fig. 2). Given that the number of patients undergoing resection in our cohort was small, we focused on the impact of adjuvant therapy on survival. Patients who received no oncological treatment (conservative and biopsy only) had a median OS of 2.4 months (95% CI 1.9–2.9 months). This, however, increased to 3.2 months (95% CI 1.2–5.2 months) if the patients also received one form of adjuvant therapy, either radiotherapy or chemotherapy. There was no statically significant difference in survival between these groups though (*p* = *0.69*). However, survival was significantly increased when patients were treated with both chemotherapy and radiotherapy (regardless of resection or biopsy) with a median OS of 6.2 months (95% CI 2.7–9.7 months) (*p* = *0.002*) (Fig. 3).

**Fig. 4:**
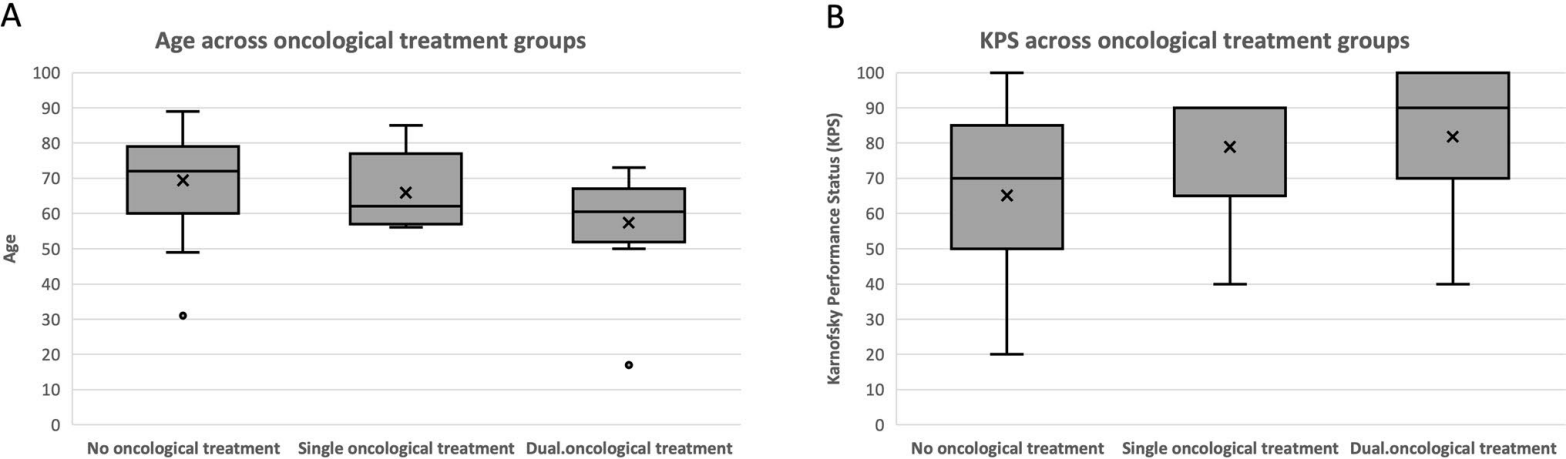
**A** Distribution of age across the treatment groups. **B** Distribution of KPS across the treatment groups

Additionally, we assessed the association of age and KPS with the oncological treatment groups. A median age across the three oncological treatment groups were 72 years old (31–89) for no oncological treatment, 62 years old (56–85) for single oncological treatment and 60.5 years old (17–73) for dual oncological treatment. The median KPS was 70 (20–100) for no oncological treatment, 90 (40–90) for single oncological treatment and 90 (40–100) for dual oncological treatment. A Kruskal–Wallis analysis demonstrated that there was a significant difference in age (*p* = *0.009*) and KPS (*p* = *0 0.015*) across the three oncological treatment groups. A pairwise analysis found that this significant difference was attributed to the older cohort and lower KPS present in the no oncological treatment group when compared to the dual oncological treatment group (age; *p* = *0.008*, KPS; *p* = *0.037*). To assess the significance of these variables as potential confounders in survival, we utilised a Cox proportional hazards model. The accuracy of the discriminating criteria was determined utilising the Omnibus test of model coefficients which showed that the predictive model was a good fit for our analysis. We found that survival was significantly impacted by the oncology treatment (*p* < 0.001), but was not significantly related to the patient’s age (*p* = 0.887) or KPS (*p* = 0.057) (Fig. 4).

## Discussion

Management of patients with bGBM presents a challenge to the neuro-oncological community. The survival is generally poor, and therefore the added value of any intervention will need to be balanced carefully against the potential side effects.

The role of resection surgery, given the bi-hemispheric nature of the tumours and the potential associated surgical morbidity, remains unclear. Chojak et al. demonstrated that resection significantly reduced the 6-month mortality rate compared with biopsy alone; however, there was no significant differences in the survival rates at 12 and 18 months [5]. More importantly, the meta-analysis did highlight that the resection and the biopsy cohorts were not adjusted for adjuvant therapy use with those in the resection cohort having a higher level of adjuvant therapy compared with the biopsy group. This may be a potential confounder which favoured survival in the resection group [5]. Boaro et al., however, showed that there was a considerable survival advantage conferred by increasing extent of resection in surgically treated bGBM patients compared to those primarily treated with chemoradiotherapy (11.5 vs. 6.3 months, respectively) [4]. The median OS for these resection patients was 11.8 months compared to 6.2 months for those that underwent biopsy followed by combined chemoradiotherapy. Notably, all patients who underwent resection received both radiotherapy and chemotherapy following surgery [4]. In our study, only 3 patients underwent resection; the low number of patients undergoing resection makes interpretation of this observation in isolation limited. As our study is a retrospective analysis, it was not powered to determine small differences between groups, and therefore limited conclusions can be drawn about the role of resection in butterfly glioma, where there is likely to be a degree of selection bias. Furthermore, within the UK, nonoperative management is favoured for bGBM patients when compared internationally. This may be related to the poor outcomes related with these bilateral lesions alongside patient presenting with a low KPS. In additional, conservative management may be largely influenced by the risks of surgical resection outweighing any potential benefits. Unwanted complications of bGBM surgery include abulia and akinetic mutism from lesion to the genu or agraphia without alexia due to damage to the splenium [7]. These complications will significantly impair quality of life in a condition where survival time is greatly shortened.

We therefore looked at the outcome of the patients based on whether they received adjuvant treatment and if so whether this was radiotherapy, chemotherapy or a combination. We found that survival was increased in patients with bGBM if they received both chemotherapy and radiotherapy compared to only one type of oncological treatment or no oncological treatment (*p* = *0.002*). These findings are in keeping with Stupp et al., who demonstrated that the use of temozolomide and radiotherapy for newly diagnosed glioblastoma resulted in a significant survival benefit [8]. Importantly we found that there was no difference in survival between those who received no oncological treatment compared to those that underwent single adjuvant treatment (*p* = *0.69*). Furthermore, despite an older cohort and a lower KPS being present in the no oncological treatment group, neither variable had an impact on overall survival when compared to the single oncological treatment and dual oncological treatment group. This would suggest that unless a patient with suspected bGBM at initial referral was deemed fit enough to receive both adjuvant radiotherapy and chemotherapy, then the added value of surgery for tissue diagnosis may be limited, especially if the diagnosis can be made radiologically with a high degree of accuracy.

Within our study, 3 patients underwent radiotherapy without histological confirmation of bGBM. There may be some reluctance to administer adjuvant treatment without a histological diagnosis, with many oncologists not prepared to commit patients to often weeks or months of intensive treatment, with associated side effects, without a definite diagnosis. In certain scenarios where the patient is of sufficiently good performance status to undergo adjuvant treatment but not fit enough for surgery or general anaesthetic (e.g. bleeding disorders or significant cardiovascular morbidity), adjuvant treatment without histological diagnosis might be considered if the radiological diagnosis is of sufficient confidence. Of course, in such cases, the lack of clinically relevant biomarkers such as MGMT might compromise the choice of the adjuvant treatment.

Importantly, the outcome of the patients remained independent of the size and location of the tumour, as well as its molecular characteristic (IDH and MGMT methylation); the only 2 significant factors were performance status and age.

### Limitations

Selection bias is a key limitation of this study. Younger patients with few co-morbidities were often selected for surgical or dual oncological management leading to slower disease progression. Furthermore, this was a retrospective study, prone to the usual limitations of data availability. Nonetheless, the findings presented do encourage future prospective studies to address the optimal management of this difficult cohort of patients.

## Conclusion

Patients who received both chemotherapy and radiotherapy following biopsy or resection of a bGBM showed greater survival when compared to single or no oncological treatment. Furthermore, there was no difference in survival between those who underwent only radiotherapy or chemotherapy compared to those that had no oncological treatment. This underlines the importance of careful evaluation of patients’ performance and fitness status to tolerate combined adjuvant therapy early in the management pathway. Advances in minimally invasive surgical techniques with less surgical morbidity might open new avenues in the management of patients with bGBM.

## Data Availability

Anonymised raw data is available.
